# Recurrent Methemoglobinemia From Over-the-Counter Medication

**DOI:** 10.7759/cureus.36014

**Published:** 2023-03-11

**Authors:** Pradeep Kumar Mada, Meagan Garibay

**Affiliations:** 1 Infectious Diseases, Comanche County Memorial Hospital, Lawton, USA; 2 Infection Control, Comanche County Memorial Hospital, Lawton, USA

**Keywords:** methylene blue infusion, local anesthetic, otc medication, acquired methemoglobinemia, benzocaine

## Abstract

A 53-year-old female with a history of rheumatoid arthritis presented with acute-on-chronic shortness of breath. She had severe lung disease secondary to chronic obstructive pulmonary disease (COPD) and recurrent pneumonia. She was found to have recurrent methemoglobinemia and presented to the hospital with refractory hypoxemia. She was given intravenous (IV) methylene blue, and transfused 2 units of packed red blood cells. Her methemoglobin levels again trended up during hospitalization and after identifying and discontinuing the offending agent, an over-the-counter (OTC) benzocaine gel, her methemoglobin level was normalized and she never had a recurrence. The severity of presentation from methemoglobinemia is increased in patients with anemia, heart disease, and pulmonary disease.

## Introduction

Methemoglobin is a form of hemoglobin that has been oxidized, altering its heme iron structure from the ferrous (Fe2+) to the ferric (Fe3+) state. Methemoglobin does not bind oxygen and cannot provide oxygen to the tissues. The normal level of methemoglobin is approximately 1% of total hemoglobin. The first reported case of topical-anesthetic-induced methemoglobinemia was documented in the 1970s [1]. The estimated overall prevalence of acquired methemoglobinemia was 0.035% during a retrospective review, and the incidence was much higher in hospitalized patients than in procedures performed outpatient (13.7 versus 0.14 cases per 10,000 procedures, respectively) [2]. The incidence of methemoglobinemia may be higher with benzocaine-containing products; nine out of 2,221 patients who received 20% benzocaine spray suffered clinically significant methemoglobinemia, while a comparison group of 22,210 patients who received 4% lidocaine spray had zero incidences of methemoglobinemia [3]. Another study estimated the incidence of methemoglobinemia at 1 in 7000 when benzocaine-containing products are used, but the exact incidence is unknown [4]. The difference in this risk may be attributed to the fact that benzocaine is a more powerful oxidizing agent than lidocaine [5]. The risk of methemoglobinemia is positively correlated with the dosage of the offending agent, and is also heightened when systemic absorption is increased, such as when the offending agent is applied to non-intact skin or mucous membranes. The risk of methemoglobinemia and, arguably, the severity of presentation can also be increased in patients who already have compromised oxygen transport, such as those with anemia, heart disease, and pulmonary disease [6].

## Case presentation

A 53-year-old female with a history of rheumatoid arthritis not on immune suppressants presented with acute-on-chronic shortness of breath. She had severe lung disease from chronic obstructive pulmonary disease (COPD) and recurrent pneumonia. She was continuously home oxygen dependent on a 4-liter nasal cannula and had a history of tobacco abuse and vaping until 2009. There was no history of other recreational drug use present. One month prior to the current admission, she was treated with intravenous (IV) levofloxacin for Stenotrophomonas pneumonia for 10 days. Her blood methemoglobin level was found to be 8% with no significant symptoms and hematology outpatient referral was placed in previous admission. She developed exertional dyspnea, pleuritic chest pain, and productive cough after completion of the antibiotic course. On arrival, her vitals were as followed: Temperature 97.6 F, Pulse 90/min, Respiration 18/min, Blood Pressure 131/87 mm of Hg, Oxygen Saturation 95% on 4-liter nasal cannula. A computerized tomography (CT) chest showed moderate to severe bilateral emphysema and multiple miliary pulmonary nodules bilaterally. Labs at presentation showed a hemoglobin of 12.3 grams per deciliter (g/dL), mean corpuscular volume (MCV) 102 femtoliter, platelet count 142,000 per microliter of blood, and white cell count was 3,400 per microliter. Ferritin, iron profile, and vitamin B-12 were normal. Antinuclear antibodies (ANA) panel was negative. On day 1 of admission, her methemoglobin was found to be 11.2% which slowly trended up to 72.8% by day 3 (Table 1).

**Table 1:** Methemoglobin level on Arterial Blood Gas Analysis

Day of admission	Methemoglobin Level %. (Reference range: 0.0-1.5%)
Day 1	11.2
Day 2	26.3
Day 3	72.8
Day 4	53.2
Day 5	39.5
Day 6	56.2
Day 7	21.1
Day 8	18.4
Day 9	22.2
Day 10	4.5
Day 11	32.7
Day 12	72.2
Day 13	73.6
Day 14	47.3
Day 15	28.4
Day 16	13.8
Day 17	3.7

We reviewed her home medication and also made sure she was not given any medication in the hospital which can cause methemoglobinemia including local anesthetics. She was intubated on day 3 for acute hypoxic respiratory failure. Pulmonology and hematology were consulted for methemoglobin level of 72.8%. She was given IV methylene blue, and transfused 2 units packed red blood cells. Her methemoglobin slowly trended down to 4.5% by day 10 and she was extubated on day 10. She also had a lung biopsy which demonstrated acute and organizing pneumonia with scattered giant cells and foreign material, most consistent with organizing aspiration pneumonia. There was no evidence of malignancy. Acid-fast bacillus (AFB) and fungal cultures were negative. Her methemoglobin level trended up to 73.6% by day 13. Hematology was consulted again and they recommended that if methylene blue was not effective, we may have to escalate care including an exchange transfusion or hyperbaric chamber and transfer the patient for a higher level of care.

At this time, we decided to go through her medication again and asked about all her medication details including over-the-counter (OTC) and herbal supplements. At that time, she mentioned she uses over-the-counter topical creams as needed for dry lips and tooth pain. One was Vaseline and another one was Orasol which has 20% benzocaine. We advised her to discontinue the cream and her methemoglobin level trended down to 3.7% by day 17. She was discharged in stable condition with a pulmonology follow-up for her severe COPD.

## Discussion

The main focus of the article is to emphasize the importance of thorough patient history which gives pivotal clues in management and avoid unnecessary treatment. Local anesthetic-induced methemoglobinemia is a well-known side effect. In our patient, we reviewed initially all physician-administered medication in the hospital and all her prescribed home medication and missed asking about over-the-counter gels. After extubation, the patient started using her Orasol for tooth pain which caused recurrent methemoglobinemia. After discontinuation of Orasol gel, she never developed methemoglobinemia. She started using Orasol gel for chronic dental pain from a botched procedure. After her hospitalization, she was referred to a dentist to fix it and strongly recommended not to use local anesthetic gels. Taking a detailed history is a vital aspect of patient care and is critical to developing a precise diagnosis and management plan. Getting a thorough history avoided unnecessary treatment and possible transfer to a higher level of care.

Clinical features of methemoglobinemia

As previously mentioned, symptoms manifest in various degrees of severity, depending on the concentration of methemoglobin in circulation [2, 6]. Since methemoglobinemia causes functional anemia and hypoxia, clinical features mimic these syndromes: tachycardia, tachypnea, headache, dizziness, fatigue, and confusion. Other signs include cyanosis (especially central cyanosis that is not responsive to oxygen therapy), dark brown discoloration of the blood that does not change color when exposed to oxygen, and low pulse oximetry readings (especially in the presence of normal arterial blood gas results). If methemoglobinemia progresses to high concentrations, arrhythmias, seizures, coma, profound acidosis, and death can occur. These signs and symptoms typically occur 20 to 60 minutes after exposure to an offending agent but can occur up to 2 hours after [7].

Pathophysiology

Methemoglobinemia is a potentially life-threatening condition in which the oxygen-carrying capacity of circulating hemoglobin is diminished due to the conversion of iron from a ferrous state to an oxidized ferric state [2]. Oxygen cannot be bound or transported by ferric iron, thus leading to a state of functional anemia and tissue hypoxia due to impaired oxygen delivery. Methemoglobinemia occurs when the concentration of methemoglobin, which normally comprises less than 1.5% of hemoglobin, exceeds this percentage. When this occurs, symptoms can manifest in varying degrees of severity based on methemoglobin concentration, but this may not always be the case [6].

Methemoglobinemia can be congenital or acquired. Congenital forms, which result from genetic defects of the cytochrome-b5 reductase enzyme or the hemoglobin structure, are rare [2]. Acquired methemoglobinemia results from exposure to substances that directly or indirectly cause hemoglobin oxidation. The most common substances known to cause acquired methemoglobinemia are the topical anesthetics benzocaine, lidocaine, and prilocaine but other substances include certain antibiotics like Clofazimine, Primaquine, Chloroquine, Dapsone, anti-parasitic agents, Quinones, Sulfonamides, amino salicylic acid, Menadione, Metoclopramide, Nitroglycerin, Phenacetin, Phenazopyridine, Rasburicase; Chemicals and environmental substances: Acetanilide (used in varnishes, rubber, and dyes); Anilines and aniline dyes (e.g., diaper and laundry marking inks, leather dyes, red wax crayons); Antifreeze; Benzene derivatives (used as solvents); Chlorates and chromates (used in chemical and industrial synthesis); Hydrogen peroxide (used as a disinfectant and cleaner); Naphthalene (used in mothballs); Naphthoquinone (used in chemical synthesis); Nitrates and nitrites (e.g., amyl nitrite, sodium nitrite, nitrate- and nitrite-containing foods, nitric oxide, well water); Nitrobenzene (used as a solvent); Paraquat (used in herbicides); resorcinol (used in resin melting and wood extraction) [7].

Treatment

Treatment of methemoglobinemia depends on the severity at the time of presentation. For mild or asymptomatic cases, supportive care, including high-flow oxygen administration and discontinuation of the offending agent, is appropriate. Moderate to severe cases, defined as symptomatic presentation with any degree of methemoglobinemia or asymptomatic presentation with methemoglobin levels greater than 30%, should receive 1-2 mg/kg of methylene blue IV over a 5-minute period [8]. Methylene blue should be given cautiously to patients who are pregnant, have documented glucose-6-phosphate dehydrogenase (G6PD) deficiency, or who are taking serotonergic medications [8]. Typically, one administration of methylene blue is sufficient to correct the methemoglobinemia, but patients should be carefully monitored for up to 12 hours after receiving the medication, as cases of rebound methemoglobinemia have occurred. Rebound methemoglobinemia can occur in 4-12 hours. Rebound may reflect the persistence of the causative agent. Additionally, in the event signs, symptoms, or blood levels do not improve within an hour of administration, the dose may need to be repeated. In the event of treatment failure, or in patients who have contraindications for methylene blue, IV high-dose ascorbic acid, blood exchange transfusion, or hyperbaric oxygen therapy may be considered [8].

U.S. Food and Drug Administration (FDA) has been closely monitoring the risk of methemoglobinemia with the use of OTC and prescription local anesthetics. FDA estimate that more than 400 cases of benzocaine-associated methemoglobinemia have been reported to them or published in the medical literature since 1971 [9-11].

## Conclusions

Taking a comprehensive patient history is an important part of the diagnostic process and can significantly influence the management plan and overall patient outcomes. Methemoglobin levels greater than 30%, should receive 1-2 mg/kg of methylene blue IV, high-flow oxygen administration, and discontinuation of the offending agent.
